# Iatrogenic type A aortic dissection after cardiac surgical or percutaneous intervention: A systematic review spanning 3 decades

**DOI:** 10.1016/j.xjon.2026.101839

**Published:** 2026-04-27

**Authors:** AlleaBelle Bradshaw, Vishnu Dontu, John Etchart, Rachel Pan, Jace C. Bradshaw, Michelle Carvajal Villalba, Shivani Shirodkar, Jennifer S. Lawton

**Affiliations:** a Division of Cardiac Surgery, Department of Surgery, Johns Hopkins University, Baltimore, Md; b Johns Hopkins University School of Medicine, Baltimore, Md; c Georgetown University School of Medicine, Washington, DC; d Department of Anesthesiology, Department of Emergency Medicine, Johns Hopkins University, Baltimore, Md

**Keywords:** aortic dissection, aortic surgery, iatrogenic

## Abstract

**Objectives:**

Iatrogenic type A aortic dissection (ITAAD) affects 0.6 to 1.0% of patients undergoing cardiac surgical or percutaneous intervention and is fatal in 20%. We performed a systematic review of ITAAD to summarize etiologies and management strategies.

**Methods:**

We searched 3 databases for reports of acute ITAAD in adults from 1990 to 2024. Retrospective cohort studies involving ITAAD after cardiac surgery, percutaneous intervention, or diagnostic catheterization were included. Case reports were analyzed separately. Data extracted included procedural cause, treatment modality, and outcome.

**Results:**

A total of 235 articles describing 860 cases of ITAAD were included. These included 19 retrospective studies (593 patients) and 216 case reports (267 patients). Of the 19 retrospective studies, 11 focused on surgical causes, 5 on percutaneous causes, and 3 included both. For surgical cases with known etiology, aortic or cardioplegia cannulation (83/135, 61%) was the most common, followed by aortic crossclamp placement (27/135, 20%). Among studies of percutaneous ITAAD (131 patients), the most common etiology was catheter trauma (106/131, 81%), and second was contrast injection (12/131, 9%). Of the 552 cases for which the method of repair was described, 438 of 552 (79%) were managed surgically, 67 of 552 (12%) percutaneously, and 47 of 552 (9%) conservatively. Overall mortality was 24% (141/593).

**Conclusions:**

We performed a systematic review of publications on ITAAD. The most common etiology for surgical ITAAD was cannulation of the aorta. ITAAD from percutaneous intervention has been increasingly reported, is commonly related to catheter manipulation, and is often successfully managed nonsurgically.

**Figure:**
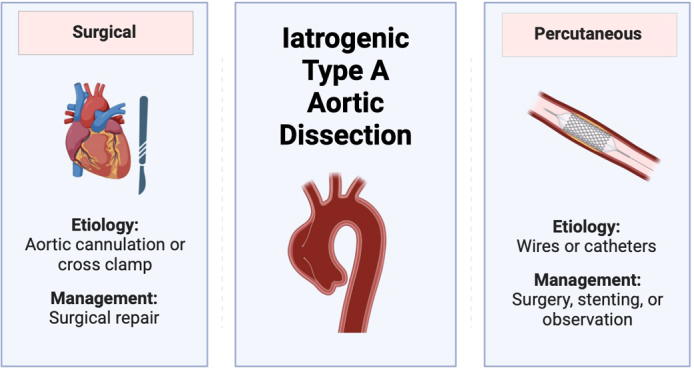
Iatrogenic type A aortic dissection occurs after surgical and percutaneous interventions.

**Central Message**

Iatrogenic type A aortic dissection arises from both surgical and percutaneous cardiac procedures, with distinct mechanisms and management strategies that influence treatment decisions and outcomes.

**Perspective**

Three decades of published studies and cases show that iatrogenic type A aortic dissection remains a rare but high-risk complication of surgical- and catheter-based interventions. Understanding procedural mechanisms and evolving management strategies may inform preventive techniques, guide early recognition, and support tailored treatments to improve outcomes.

Among patients with type A aortic dissection who are treated surgically, approximately 4% of cases are iatrogenic.^1^ The incidence of iatrogenic type A aortic dissection (ITAAD) after cardiac surgery is approximately 0.06% and is associated with a high mortality rate.^2^^,^^3^ Similarly, the incidence of ITAAD after percutaneous intervention is 0.02 to 0.06%, with comparably poor outcomes.^4^, ^5^, ^6^ Despite advances in surgical and endovascular techniques, iatrogenic dissections continue to occur and remain associated with substantial mortality.^7^

Many retrospective reports and case series summarize ITAAD etiology and management. However, these often examine ITAAD after surgery or percutaneous intervention, and the two are infrequently evaluated together. Summarizing all reported studies and cases together may offer valuable insight that is not present in isolated surgical or procedural reviews.

We performed a systematic review of published cases and retrospective studies of ITAAD to characterize etiologies and management strategies. Given the relative infrequency of ITAAD, summarizing more than 3 decades of published studies can provide insight into patterns, highlight knowledge gaps, and inform preventive and therapeutic approaches in contemporary practice.

## Methods

Institutional review board approval and informed consent were not applicable. A systematic literature search using the PubMed, Embase, and Web of Science databases was performed. Search terms were “iatrogenic aortic dissection” and “intraoperative aortic dissection,” with variations in spelling and indexing adapted for each database. Additional studies were identified through screening reference lists of included articles. All cases and studies of ITAAD published between 1990 and 2024 that included data about the etiology and management of the dissection were considered. Studies that were not in English or did not include data about etiology and management were excluded. Two reviewers screened each article to determine eligibility.

Study or patient characteristics documented included first author, year of publication, study period, country, study design, and number of patients with ITAAD. Eligible patients were adults ≥18 years who had undergone cardiac surgery or percutaneous cardiac intervention (PCI; eg, coronary angiography, PCI, transcatheter aortic valve intervention, or other cardiac instrumentation) who developed an aortic dissection during the procedure or within 30 days. Articles were excluded if patients had iatrogenic abdominal aortic dissection (type B or without ascending or aortic arch involvement) or if the thoracic aortic dissection resulted from a retrograde extension of a previous descending aortic dissection.

Demographic variables were collected including age, sex, type of initial surgery or procedure, and indication for initial surgery or procedure. Additional variables included vessels involved, dissection origin, method of diagnosis, and management. Variables that were inconsistently reported and thus not analyzed included the presence of aortic calcification, baseline medical history, previous cardiac procedures, and outcomes other than mortality. The only outcome included was mortality, which was defined according to each study (in-hospital, 30-day, or operative).

Data from retrospective studies were summarized descriptively using counts and percentages for categorical variables and pooled averages when appropriate. Because of heterogeneity in study design, reporting methods, and available variables across studies, a formal meta-analysis was not performed. Comparisons of management strategies (surgical, percutaneous, or conversative) according to procedural cause (surgical vs percutaneous) were evaluated using χ^2^ tests.

## Results

**Figure 1:**
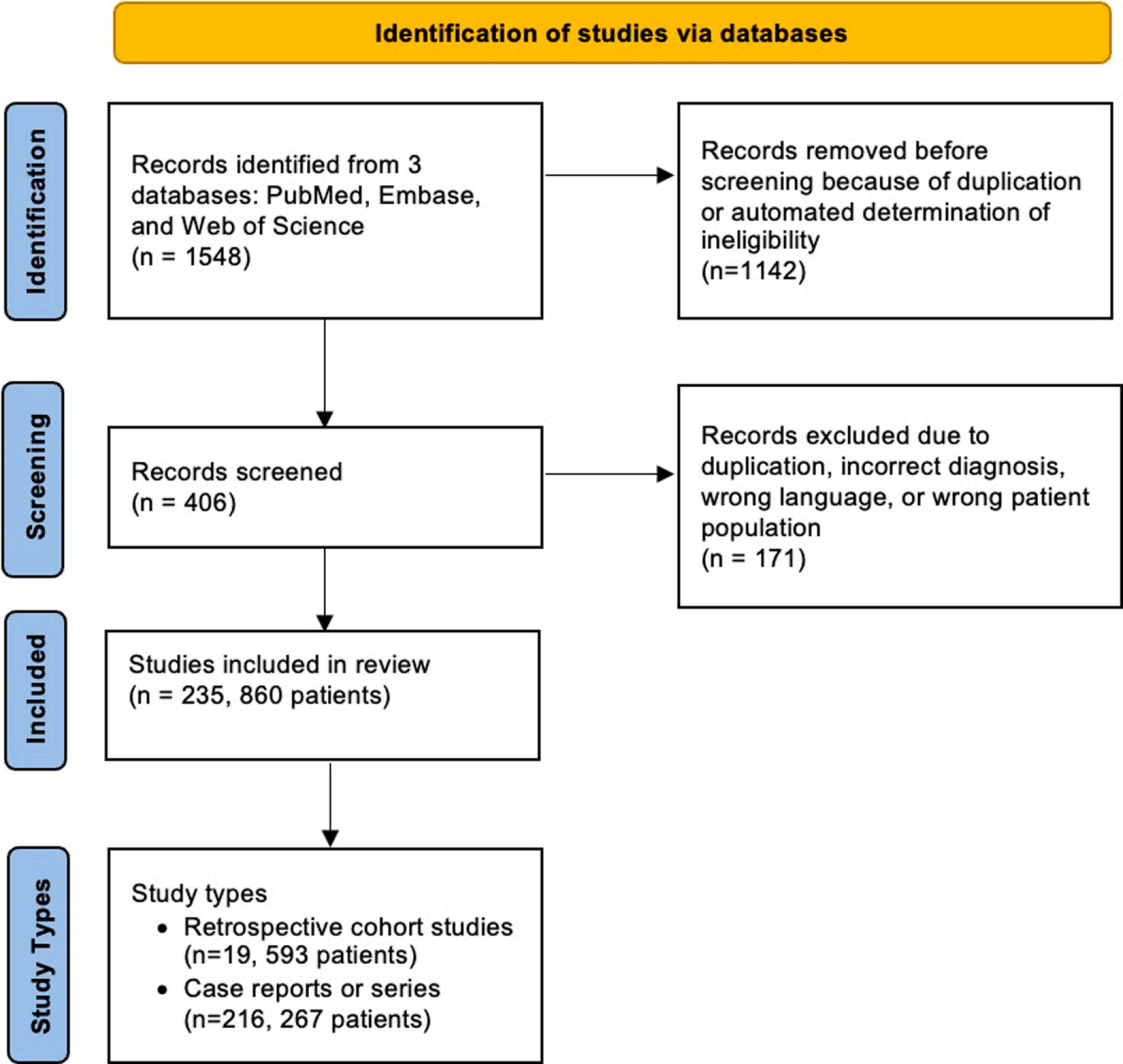
Preferred Reporting Items for Systematic reviews and Meta-Analyses diagram. After screening, 235 articles describing 860 cases were included. These included 19 retrospective studies (593 patients) and 216 case reports or case series (267 patients).

**Figure 2:**
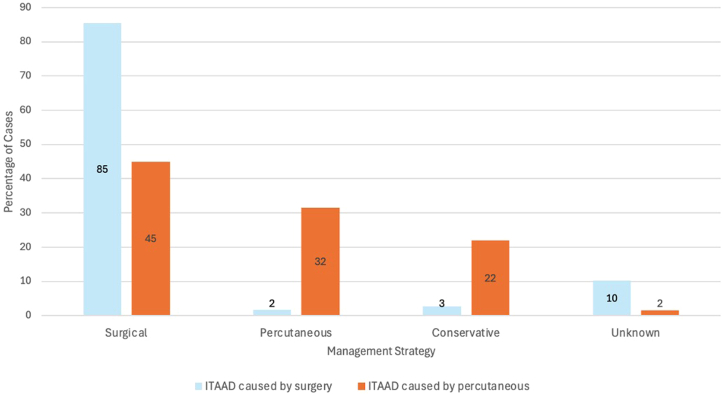
Management strategy for ITAAD on the basis of procedural cause of dissection. Percentages (shown in *columns*) were calculated by dividing the number of cases in a given category (n) by the total number of cases (860). *ITAAD*, Iatrogenic type A aortic dissection.

After screening, 235 articles describing 860 cases of ITAAD were included. These included 19 retrospective studies (593 patients) and 216 case reports or series (267 patients) (Figure 1). In the retrospective studies, 46% (272/593) of cases were ITAADs caused by surgery. In the case reports, only 37% (99/267) of cases were caused by surgery. Of the 860 total cases of ITAAD, 412 (48%) occurred during or after surgical intervention, 438 (51%) occurred from percutaneous interventions, and 10 (1%) were from unspecified procedures. Among all reported cases, the most common management overall was surgical, but nonsurgical management was most common when the dissection occurred from a percutaneous intervention (*P* < .001) (Figure 2).

### Retrospective Cohort Studies

**Figure 3:**
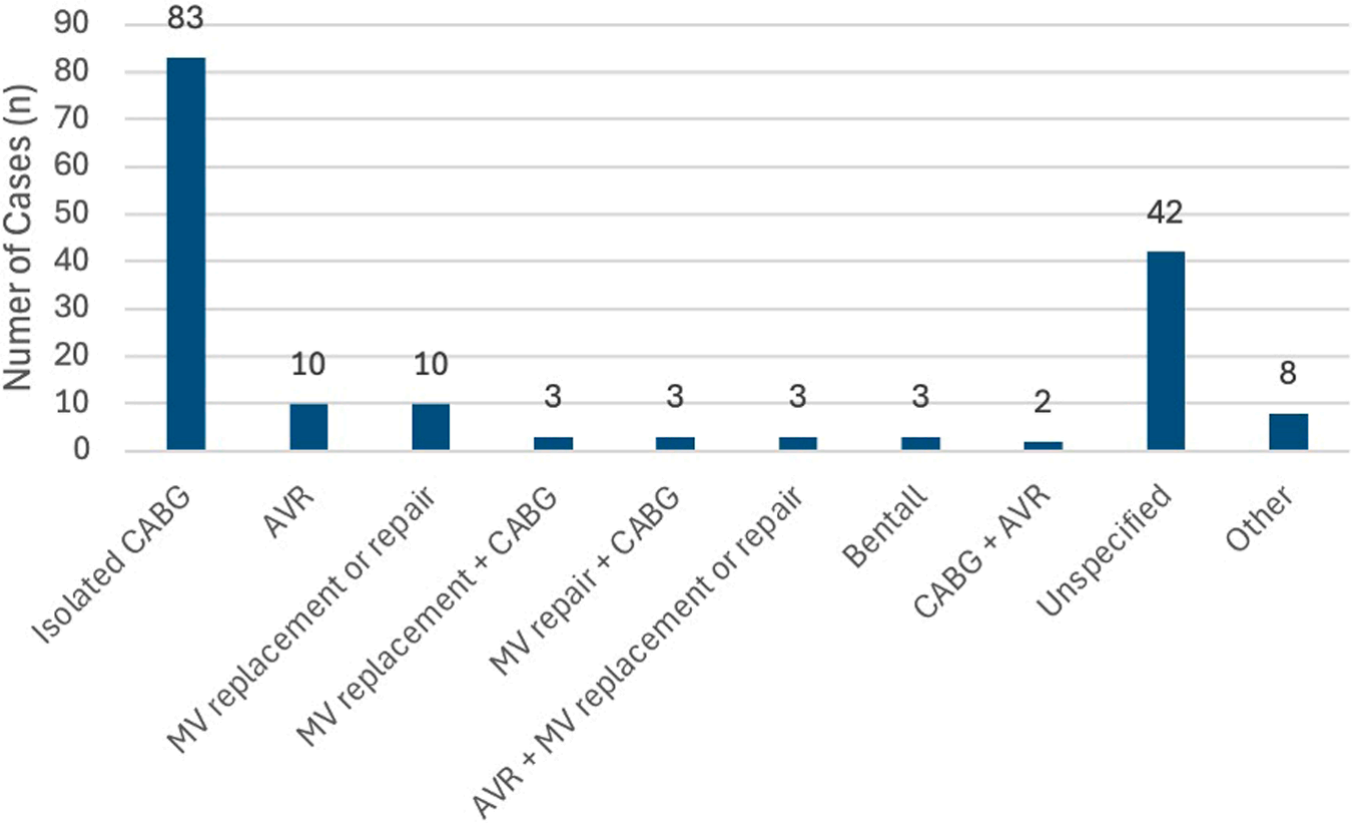
Cardiac surgery case types associated with iatrogenic type A aortic dissection in published retrospective cohort studies. Among 11 retrospective studies describing iatrogenic type A aortic dissection published from 1990 to 2024, surgical case types were reported. *CABG*, Coronary artery bypass grafting; *AVR*, aortic valve replacement; *MV*, mitral valve.

Among retrospective studies, 11 of 19 (58%) focused on surgical causes, 5 (26%) on percutaneous causes, and 3 (16%) included both. Ten of the 11 surgical studies (135/167 patients) reported an etiology. Aortic or cardioplegia cannulation (83/135, 61%) was the most common etiology, and aortic cross-clamp was second (27/135, 20%) (Table 1, Table E1).^5^^,^^8^, ^9^, ^10^, ^11^, ^12^, ^13^, ^14^, ^15^, ^16^, ^17^, ^18^, ^19^, ^20^, ^21^, ^22^, ^23^, ^24^, ^25^ Case type was reported in 125 of 167 (75%) patients in the surgical ITAAD studies. Isolated coronary artery bypass grafting was the case type most frequently reported (83/125, 66%) (Figure 3).

**Table 1:** Retrospective studies of iatrogenic type A aortic dissection after cardiac, percutaneous, or combined procedures

First author, year	ITAAD incidence	Population	Etiology			Management			
			Cannulation	Crossclamp	Catheter	Surgery	Catheter	Conservative	Mortality
Surgical and percutaneous									
Rylski 2013^8^	100∗	Surgical or postinterventional (catheterization, EVAR, TAVR) GERAADA database	NK	NK	47 (47)	100 (100)	0	0	16 (16.0)
Von Aspern 2021^9^	92/87,524 (0.11)	ITAADs after cardiac or interventional procedures, excluded if treated non-surgically Single center (Germany)	41 (45)	0	28 (30)	92 (100)	0	0	41 (44.7)
Biancari 2022^10^	103∗	ITAADs after cardiac or interventional procedures, excluded if treated nonsurgically ERTAAD database	NK	NK	64 (62)	103 (100)	0	0	31 (30.1)
Percutaneous									
Boukhris 2015^11^	8/956 (0.84)	PCI for CTO Single center (Tunisia)	0	0	8 (100)	0	8 (100)	0	1 (12.5)
Nunez-Gil 2015^5^	74/108,083 (0.07)	Diagnostic or therapeutic PCI Multicenter (Europe)	0	0	74 (100)	3 (4)	35 (47)	36 (49)	2 (2.7)
Baumann 2017^12^	8/3600 (0.22)	PCI Single center (Germany)	0	0	8 (100)	0	5 (63)	3 (38)	0
Huenges 2017^13^	14∗	Diagnostic or therapeutic PCI Surgically treated pts included Comparison with noniatrogenic TAAD Single center (Germany)	0	0	14 (100)	14 (100)	0	0	7 (50.0)
Kostantinis 2023^14^	27/12,117 (0.22)	PCI for CTO PROGRESS-CTO registry	0	0	27 (100)	0	19 (70)	8 (30)	0
Surgical									
Still 1992^15^	24/14,877 (0.16)	Cardiac surgery on bypass Single center (US)	10 (42)	15 (63)	0	24 (100)	0	0	6 (25.0)
Ruchat 1998^16^	10/8624 (0.12)	Cardiac surgery on bypass Single center (Switzerland)	7 (70)	0	0	9 (90)	0	0	3 (30)
Savolainen 2003^17^	9/9118 (0.10)	Cardiac surgery on bypass Single center (Switzerland)	6 (67)	0	0	8 (89)	0	0	3 (33.3)
Fleck 2006^18^	7/3000 (0.23)	Cardiac (5), ascending aortic (1), or lung transplant (1) surgery Single center (Austria)	7 (100)	0	0	7 (100)	0	0	3 (42.9)
Ketenci 2008^19^	21/10,130 (0.20)	Conventional CABG surgery Single center (Turkey)	5 (24)	9 (43)	0	21 (100)	0	0	10 (47.6)
Hurt 2008^20^	32/12,907 (0.25)	Cardiac surgery Single center (US)	NK	NK	0	NK	NK	NK	8 (25.0)
Hwang 2010^21^	10/3421 (0.29)	Cardiac surgery on bypass Single center (South Korea)	9 (90)	0	0	10 (100)	0	0	4 (40.0)
Stamou 2013^22^	11/15,900 (0.07)	Cardiac surgery with ITAAD diagnosed intraoperatively Multicenter (US) Comparison with spontaneous TAAD	4 (36)	0	0	11 (100)	0	0	3 (27.3)
Narayan 2015^23^	7/15,144 (0.05)	Cardiac surgery with ITAAD diagnosed intraoperatively Single center (UK) Comparison with spontaneous TAAD	5 (71)	2 (29)	0	NK	NK	NK	2 (28.6)
Shea 2019^24^	15/23,275 (0.06)	Cardiac surgery Single center (US)	11 (73)	1 (7)	0	15 (100)	0	0	1 (6.7)
Wang 2022^25^	21/23,143 (0.09)	Cardiac or aortic surgery (all with bypass except one) Single center (China)	19 (90)	0	0	21 (100)	0	0	2 (9.5)

Etiologies categorized as “cannulation” include both arterial and cardioplegia cannulation for presentation in this table. Etiologies categorized as “catheter” include percutaneous causes of dissection (catheter trauma, stent, etc). Dissection caused by less-common etiologies are not shown. The ITAAD incidence (second column) indicates the ITAAD (numerator), the total procedures (denominator), and the percentage of patients who had an ITAAD in that study. Values shown as n (%). *ITAAD*, Iatrogenic type A aortic dissection; *EVAR*, endovascular aortic repair; *TAVR*, transcatheter aortic valve replacement; *GERAADA*, German Registry of Acute Aortic Dissection Type A; *NK*, not known; *ERTAAD*, European Registry of Type A Aortic Dissection; *PCI*, percutaneous coronary intervention; *CTO*, chronic total occlusion; *TAAD*, type A aortic dissection; *CABG*, coronary artery bypass grafting.

∗ Total number of procedures (denominator) not reported in the study.

**Figure E1:**
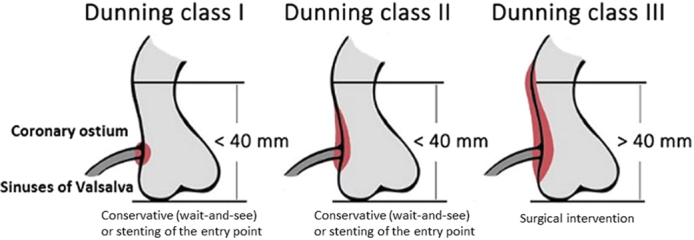
Dunning classification for aortocoronary dissection. The Dunning classification categorizes coronary artery dissections that extend into the aortic root on the basis of the extent of aortic involvement.^6^ Reprinted with permission.^12^https://creativecommons.org/licenses/by/4.0/.

Of the 5 studies focusing on ITAAD after percutaneous intervention (131 patients), the Dunning classification was described in 4 studies (104 patients). The Dunning classification categorizes aortocoronary dissection as Dunning I, confined to the sinus of Valsalva; Dunning II, extension <40 mm; and Dunning III, extension ≥40 mm (Figure E1). Dunning I was most common (52/104, 50%), followed by Dunning II (31/104, 30%), and Dunning III (19/104, 18%). The most common percutaneous procedure causing ITAAD was PCI (110/131, 84%), followed by diagnostic angiography (21/131, 16%). The most common specific etiology was catheter trauma (106/131, 81%), and second was contrast injection (12/131, 9%). Four studies (117 patients) described the most common coronary location of the dissection origin, and the right was more common (82/117, 70%) than the left (Table 2).^5^^,^^11^, ^12^, ^13^, ^14^

**Table 2:** Procedural details from iatrogenic aortocoronary dissections after percutaneous cardiac procedures reported in retrospective studies

Study	n	Dunning classification			Procedure		Origin		Etiology		
		I	II	III	Diagnostic	PCI	Right coronary	Left coronary	Catheter trauma	Wire	Contrast
Boukhris 2015^11^	8	2 (25)	6 (75)	0	0	8 (100)	7 (88)	1 (12)	6 (75)	0	1 (12)
Nunez-Gil 2015^5^	74	45 (61)	12 (16)	15 (20)	16 (22)	58 (78)	42 (57)	30 (41)	68 (92)	2 (2.7)	0
Baumann 2017^12^	8	3 (38)	1 (13)	4 (50)	0	8 (100)	7 (88)	1 (12)	5 (63)	1 (12)	0
Huenges 2017^13^	14	2 (14)	12 (86)	0	5 (36)	9 (64)	NK	NK	14 (100)	0	0
Kostantinis 2023^14^	27	NK	NK	NK	0	27 (100)	26 (96)	1 (4)	13 (48)	0	11 (41)

Variables collected included Dunning classification, initial procedure performed, origin of dissection, and suspected etiology. The Dunning classification indicates the extent of dissection. The second column (“n”) shows the total number of ITAADs in the study. *PCI*, Percutaneous coronary intervention; *NK*, not known; *ITAAD*, iatrogenic type A aortic dissection.

In retrospective studies, the method of repair was described in 552 cases. Of these, 438 of 552 (79%) were managed surgically, 67 of 552 (12%) were managed percutaneously, and 47 of 552 (9%) were managed conservatively (watch-and-wait) (Table 1). Specific surgical treatment methods were inconsistently reported.

Mortality rates in retrospective studies ranged from 7% to 43% among studies with surgical causes and 0% to 50% for studies with percutaneous causes of ITAAD (Table 1). Overall weighted mortality for retrospective studies was 24% (141/593). Of all deaths reported in retrospective studies, 32 reported a cause. The most commonly reported cause was cardiogenic shock or ventricular failure (16/32, 50%), followed by multiorgan failure (7/32, 22%) (Table E2).

### Case Reports

Among case reports, 99 of 267 patients (37%) developed ITAAD after cardiac surgery and 168 of 267 (63%) after percutaneous procedures, such as coronary angiography, PCI, or transcatheter aortic valve replacement (TAVR). The most common etiology for surgical cases was aortic cannulation (43/99, 43%) or crossclamp (10/99, 10%). Management included open surgery (121/267, 45%), percutaneous intervention (78/267, 29%), conservative (60/267, 22%), and unknown/not stated (8/267, 3%). Among those who underwent surgical management, the most common surgery performed was an ascending aorta replacement or repair (42/121, 35%). Other operations performed included coronary artery bypass grafting only (15/121, 12%), ascending aorta plus hemiarch repair or replacement (7/121, 6%), and Bentall (6/121, 5%). Additional analysis was performed for specific variables from case reports (Table E3).

**Figure 4:**
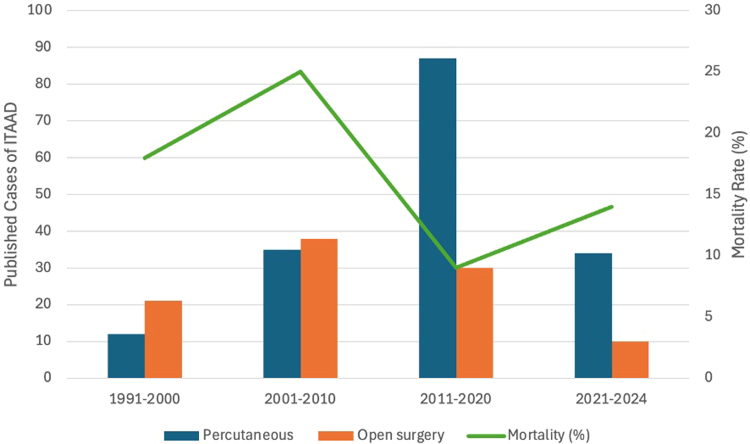
Case reports describing iatrogenic aortic dissection after surgical or percutaneous cardiac intervention between 1990 and 2024. These case numbers and mortality rate represent only published ITAAD cases, which may not reflect all cases. *ITAAD*, Iatrogenic type A aortic dissection.

Etiologies and management strategies were evaluated by decade to assess for trends in occurrence over time. Between 1991 and 2000, most published ITAAD cases had a surgical etiology, whereas after 2001 the majority had a percutaneous etiology (Table 3, Figure 4). Overall mortality including all 267 case reports was 16% (42/267).

**Table 3:** Case reports describing iatrogenic aortic dissection after surgical or percutaneous cardiac intervention over 4 time periods

Variable	Totals	1991-2000	2001-2010	2011-2020	2021-2024
Articles	216	16	59	110	31
Patients	267	33	73	117	44
Initial procedure					
Percutaneous	168 (63)	12 (36)	35 (48)	87 (74)	34 (77)
Surgery	99 (37)	21 (64)	38 (52)	30 (26)	10 (23)
Vessels involved					
Aorta only	106 (40)	21 (63)	32 (44)	37 (32)	16 (36)
Aorta + coronary	151 (56)	11 (33)	40 (55)	76 (65)	24 (55)
Aorta + innominate	7 (3)	1 (3)	0 (0)	4 (3)	2 (5)
Aorta + coronary + innominate	1 (0.4)	0 (0)	0 (0)	0 (0)	1 (2)
Aorta + subclavian	1 (0.4)	0 (0)	1 (1)	0 (0)	0 (0)
Aorta + R iliac	1 (0.4)	0 (0)	0 (0)	0 (0)	1 (2)
Etiology					
Cannulation site	43 (16)	15 (45)	16 (22)	10 (9)	2 (5)
Cross-clamp site	10 (4)	0 (0)	4 (5)	3 (3)	2 (5)
CABG anastomosis	6 (2)	2 (6)	4 (5)	0 (0)	0 (0)
Percutaneous	140 (52)	13 (39)	34 (47)	66 (56)	28 (64)
Not known	68 (25)	3 (9)	15 (21)	38 (32)	12 (27)
Management					
Surgery	121 (45)	22 (67)	43 (59)	41 (35)	15 (34)
Percutaneous	78 (29)	10 (30)	18 (25)	37 (32)	13 (30)
Conservative	60 (22)	1 (3)	11 (15)	33 (28)	15 (34)
Not stated	8 (3)	0 (0)	1 (1)	6 (5)	1 (2)
Mortality					
Prior to repair	10 (4)	1 (3)	4 (5)	2 (2)	3 (7)
Operative mortality	11 (4)	2 (6)	4 (5)	3 (3)	2 (5)
Postoperative mortality	21 (8)	4 (12)	10 (14)	6 (5)	1 (2)

Data are presented as n (%). Percentages are calculated as n divided by the total number of patients (third row) within the given period. *CABG*, Coronary artery bypass grafting.

## Discussion

The primary aim of this systematic review was to summarize published cases and studies of ITAAD, focusing on etiology and management strategies reported over the last 34 years. Patterns emerged in both mechanism and treatment: ITAAD after cardiac surgery was most often associated with aortic cannulation or crossclamping and typically required operative repair. ITAAD after percutaneous intervention was most often catheter-related and was more frequently managed nonsurgically compared with surgery-related ITAAD in reported cases.

The largest study to date of surgery-related ITAAD was by Williams and colleagues,^2^ which evaluated a cohort of 1294 patients from the Society of Thoracic Surgeons database. It provided insight into the risk factors and mortality rate of this complication, but it lacked information on etiology (eg, cross-clamp or aortic cannulation) and specific operative management strategies. More recently, Liu and colleagues contributed the only large database study to date on catheter-related ITAAD (n = 971), also using the Society of Thoracic Surgeons database. This study provided insight into these patients and outcomes but was limited by only including patients who underwent operative repair. By collating both etiologies of ITAAD, this review highlights both distinct procedural risk factors and differences in therapeutic approaches that are relevant to current practice.^26^

Identifying common etiologies is important, because it can stimulate changes in technique or device design that may improve outcomes. The aortic cannulation site has been the most common site of surgical iatrogenic dissection since at least the 1970s.^27^ The cross-clamp site is the next most common. Cases involving a side-biting or partial clamp have been hypothesized as being associated with increased risk of dissection,^28^^,^^29^ although some studies have indicated no increased risk with this technique.^2^^,^^23^ With PCI, barotrauma and manipulation of the guiding catheter are 2 of the common causes of dissection.^30^ Certain procedures, such as chronic total occlusion interventions^11^^,^^14^ or TAVR,^9^^,^^31^ appear to be associated with a particularly high risk of ITAAD. TAVR was approved in the United States in 2011, and its use has only increased, which may partially account for the increase in ITAAD that is associated with percutaneous interventions seen from 2011 to 2020 compared with the previous decade. In this study, there were 17 reports of ITAAD after TAVR, with 15 occurring after 2011.

Regardless of the specific cause of ITAAD, the principles of management share common goals. As stated by Gennari and colleagues,^32^ “the goal in the treatment of ITAAD should be closing the intimal tear as quickly as possible to prevent the progression of dissection” Many dissections identified during PCI can be treated intraprocedurally with stenting, although many patients have clinically significant residual dissections.^30^ The determinant of whether surgery is required may depend on the extent of the dissection. This review of the literature finds that many clinicians are treating Dunning I and II nonsurgically, while Dunning III are being treated surgically (Figure 4).^12^ However, current guidelines still recommend surgery for all acute type A aortic dissections.^33^ When patients with ITAAD after percutaneous intervention require surgery, they tend to have more extensive dissections and require more complex repairs compared with surgical patients.^8^ A standardized algorithm was developed by Shea and colleagues,^24^ and is worth consideration for wider adoption, given their low mortality rate following ITAAD.

Surgical repair is the most common management strategy for ITAAD, although cases related to percutaneous procedures may sometimes be treated percutaneously or conservatively. In this review, case reports more frequently described nonoperative management compared to retrospective studies, likely reflecting a publication bias favoring successful use of nontraditional strategies. Most case reports describing operations to manage ITAAD include ascending aorta replacement or repair, which was similar to what was published in retrospective studies that included operative data.^10^^,^^24^ A limitation of several studies was that they only identified patients who underwent surgical intervention for ITAAD and thus missed patients who were managed conservatively, percutaneously, or who died before intervention could be performed.^8^, ^9^, ^10^^,^^13^^,^^25^

This study has limitations. The heterogeneity of published data precluded meta-analysis, as reporting of variables such as comorbidities, operative details, and long-term outcomes was inconsistent. The interpretability of the mortality rates, in particularly, is limited. Retrospective studies often lacked granular procedural information, while case reports are subject to publication bias, particularly underreporting of fatal cases. In addition, database studies frequently omit etiology-specific data, limiting mechanistic insight. Despite these limitations, this review synthesizes ITAAD, emphasizing the contexts in which ITAAD is likely to occur, management strategies most often used, and the persistently high mortality.

## Conclusions

This systematic review identified trends in published literature on this high-risk complication over a 34-year period. The literature shows that opportunities remain for technical and device innovation to reduce the incidence of these complications. Although surgically caused ITAAD is still treated surgically, there is accumulating literature that many percutaneously caused ITAAD cases can be managed nonsurgically. Further study and development of specific guidelines may help to establish best practices.

## Conflict of Interest Statement

The authors reported no conflicts of interest.

The *Journal* policy requires editors and reviewers to disclose conflicts of interest and to decline handling or reviewing manuscripts for which they may have a conflict of interest. The editors and reviewers of this article have no conflicts of interest.

## Figures and Tables

**Table E1:** Additional data for retrospective studies of ITAAD

Study	Mortality definition	Time to diagnosis	Malperfusion	Cause of death	Cardioplegia vs aortic cannulation
Surgical and percutaneous					
Rylski 2013^8^	30 d	Symptoms to surgery: average 5 h (shorter in surgical compared with percutaneous)	Neuro (2/100) Coronary (25/100) Limb (2/100) Renal (1/100)	Not described	Not described
Von Aspern 2021^9^	In-hospital or 30 d	Surgical cause: intraoperative diagnosis in 53/64 (83%), postoperative diagnosis in 11/64 (17%). Timing not described for percutaneously caused ITAAD.	Coronary (30/92, 33%)	Not described	Aortic (24/64, 37%) vs cardioplegia (17/64, 27%)
Biancari 2022^10^	In-hospital	Symptoms to surgery: median 10.5 h Primary procedure to surgery for ITAAD: median 1.5 d	Cerebral (10/103) Spinal (2/103) Renal (6/103) Mesenteric (3/103) Peripheral (8/103)	Not described	Not described
Percutaneous					
Boukhris 2015^11^	Not defined. One patient died 12 d after index procedure.	Not quantified, but “diagnosis occurred during procedure or immediately after.”	Not explicitly reported. (Report implies that no malperfusion syndromes were diagnosed.)	Not described	NA
Nunez-Gil 2015^5^	Not defined. Two patients died during follow-up. Median follow-up 51 mo.	ITAAD was identified during the procedure in all cases.	Not reported.	One patient died due to MI and 1 patient died due to left ventricular dysfunction.	NA
Baumann 2017^12^	Not defined. No patients died.	Of the 8 patients, 7 were diagnosed with ITAAD during angiography. One was diagnosed on CT immediately after.	Not reported.	No patients died.	NA
Huenges 2017^13^	30 d	Time frame not known in all cases, but 12/14 (86%) diagnosed during angiography, and 2 diagnosed postprocedure by CT.	Myocardial (8/14, 57%) Limb (1/14, 7%) Neuro (1/14, 7%)	Not reported.	NA
Kostantinis 2023^14^	In-hospital	Not reported. (Intraprocedural recognition for most cases is implied.)	Not reported.	No patients died.	NA
Surgical					
Still 1992^15^	Operative/in-hospital	Intraoperative diagnosis (20/24, 83%) vs postoperative diagnosis (4/24, 17%)	Not quantified. (Report mentions multiple pts with malperfusion.)	Four died intraoperatively from ventricular failure. Two died postoperatively (from aortic rupture or multiorgan failure).	Not described.
Ruchat 1998^16^	In-hospital	Intraoperative diagnosis (7/10, 70%) vs postoperative diagnosis (3/10, 30%)	Cerebral (2/10, 20%)	Two died from anoxic brain injury. One died from cardiogenic shock.	Aortic cannulation caused 60% (6/10). No cases attributed to cardioplegia cannulation.
Savolainen 2003^17^	In-hospital	Intraoperative diagnosis (6/9, 67%) vs postoperative diagnosis (3/9, 33%)	Limb (1/9, 11%)	Specific causes of death were not reported. Two patients died from cardiogenic shock. One cause was not described.	Aortic cannulation caused 6/9 (67%). No cases attributed to cardioplegia cannulation.
Fleck 2006^18^	In-hospital	All diagnosed intraoperatively: Beginning of bypass (4/7, 57%) vs end of operation on echocardiography (3/7, 43%)	Not reported.	One died from heart failure, 1 from multiorgan failure, and 1 from respiratory failure.	Aortic cannulation caused 3/7 (43%). Cardioplegia cannulation caused 1/7 (14%).
Ketenci 2008^19^	In-hospital	Intraoperative (19/21, 90%) vs early postoperative (2/21, 10%)	Limb (2/21, 9%)	Five died intraoperatively from cardiac failure. Two died postoperatively due to bleeding.	Aortic cannulation caused 5/21 (24%). Cardioplegia cannulation is not mentioned as a cause.
Hurt 2008^20^	In-hospital	Not reported.	Not reported.	Not reported.	Not reported.
Hwang 2010^21^	Operative	All diagnosed intraoperatively.	Cerebral (1/10, 10%) Limb (1/10, 10%)	Three died due to multiorgan failure. One died due to hypoxic brain injury.	Ascending aortic cannulation (4/10, 40%). Aortic root cannulation (2/10, 20%)
Stamou 2013^22^	Operative	All diagnosed intraoperatively.	Not reported.	Not reported.	Aortic cannulation (4/11, 36%). Aortic root vent (4/11, 36%).
Narayan 2015^23^	In-hospital	All diagnosed intraoperatively.	Not reported.	One patient died from cardiac failure. One died from multiorgan failure.	Ascending aortic cannulation (3/7, 43%). Cardioplegia cannulation (1/7, 14%).
Shea 2019^24^	In-hospital	Intraoperative diagnosis (14/15, 93%) vs postoperative diagnosis (1/15, 7%).	None identified.	One patient died from multiorgan failure.	Aortic cannulation (10/15, 67%). Cardioplegia cannulation (2/15, 13%).
Wang 2022^25^	In-hospital	All diagnosed intraoperatively.	Coronary (1/21, 5%)	Two patients died of aortic rupture postoperatively.	Aortic cannulation (10/21, 47%). Cardioplegia cannulation (38%).

Additional data for retrospective studies of (ITAAD) are shown, including the mortality as defined in each study, time to diagnosis, malperfusion syndromes, causes of death, and specific etiologies related to cannulation. *ITAAD*, Iatrogenic acute type A aortic dissection; *CT*, computed tomography; *MI*, myocardial infarction.

**Table E2:** Causes of death reported in retrospective studies of ITAAD

Cause of death	n (%)
Deaths from ITAAD in which cause of death was described	32 (100)
Cardiogenic shock or ventricular failure	16 (50)
Multiorgan failure	7 (22)
Aortic rupture	3 (9)
Anoxic brain injury	3 (9)
Postoperative bleeding	2 (6)
Respiratory failure	1 (3)

Retrospective studies reporting ITAAD were reviewed. Of the studies included, there were 32 patients in whom a cause of death related to ITAAD was described. Causes are shown. “Cardiogenic shock” includes patients who died from myocardial infarction, ventricular failure, or otherwise undescribed cardiogenic shock. *ITAAD*, Iatrogenic acute type A aortic dissection.

**Table E3:** Summary of reported data from case reports of ITAAD

Case reports	Value
Total cases	267
Age, y	66 ± 16
Female	139 (52)
Initial procedure	
PCI	113 (42)
CABG	44 (16)
Diagnostic catheterization	35 (13)
Valve surgery	21 (8)
TAVR	17 (6)
Vessels affected	
Aorta only	106 (40)
Aorta + RCA	104 (39)
Aorta + LCA	38 (14)
Aorta + RCA + LCA	9 (3)
Aorta + Innominate artery	7 (3)
Diagnosis	
Angiography	100 (37)
CT angiography	60 (22)
Echocardiography	46 (17)

In this systematic review of ITAAD, there were 267 published cases identified of ITAAD occurring after either surgical or percutaneous interventions. Variables such as age, sex, initial procedure performed, vessels affected by dissection, and method of dissection are summarized. Data are presented as n (%). The percentage is calculated as *n* divided by the total number of cases (267). *ITAAD*, Iatrogenic acute type A aortic dissection; *PCI*, percutaneous coronary intervention; *CABG*, coronary artery bypass grafting; *TAVR*, transcatheter aortic valve replacement; *RCA*, right coronary artery; *LCA*, left coronary artery; *CT*, computed tomography.
